# Serum Amyloid A Induces NLRP-3-Mediated IL-1β Secretion in Neutrophils

**DOI:** 10.1371/journal.pone.0096703

**Published:** 2014-05-20

**Authors:** Kiyoshi Migita, Yasumori Izumi, Yuka Jiuchi, Hideko Kozuru, Chieko Kawahara, Minoru Nakamura, Tadashi Nakamura, Kazunaga Agematsu, Junya Masumoto, Michio Yasunami, Atsushi Kawakami, Katsumi Eguchi

**Affiliations:** 1 Department of Rheumatology and Clinical Research Center, Nagasaki Medical Center, Omura, Nagasaki, Japan; 2 Department of Rheumatology, Shinto Kumamoto Hospital, Kumamoto, Japan; 3 Department of Infection and Host Defense, Graduate School of Medicine, Shinshu University, Matsumoto, Nagano, Japan; 4 Department of Pathology, Ehime University Graduate School of Medicine and Proteo-Science Center, Toon, Ehime, Japan; 5 Institute of Tropical Medicine (NEKKEN), Nagasaki University, Nagasaki University, Nagasaki, Japan; 6 Department of Rheumatology, Nagasaki University Hospital, Nagasaki, Japan; 7 Department of Rheumatology, Sasebo City General Hospital, Sasebo, Nagasaki, Japan; Toho University School of Medicine, Japan

## Abstract

**Background/Aims:**

Serum amyloid A (SAA) is an acute phase reactant with significant immunological activities, including effects on cytokine synthesis and neutrophil chemotaxis. Neutrophils can also release cytokines with proinflammatory properties. IL-1β is a key proinflammatory cytokine, the secretion of which is controlled by inflammasome. We investigated the proinflammatory effects of SAA *in* *vitro* in relation to the NLRP3 inflammasome in neutrophils.

**Methodology/Principal Findings:**

Human neutrophils isolated form healthy subjects were stimulated with serum amyloid A (SAA). The cellular supernatants were analyzed by western blot using anti-IL-1β or anti-caspase-1 antibodies. IL-1β or Nod-like receptor family, pyrin domain containing 3 (*NLRP3*) mRNA expressions were analyzed by real-time PCR or reverse transcription-PCR (RT-PCR) method. SAA stimulation induced pro-IL-1β mRNA expression in neutrophils. Furthermore, SAA engaged the caspase-1-activating inflammasome, resulting in the production of active IL-1β. SAA-induced pro-IL-1β expression was marginally suppressed by the Syk specific inhibitor, R406, and SAA-induced pro-IL-1β processing in neutrophils was prevented by R406. Furthermore, SAA-induced *NLRP3* mRNA expression was completely blocked by R406. Analysis of intracellular signaling revealed that SAA stimulation activated the tyrosine kinase Syk and mitogen-activated protein kinase (MAPK).

**Conclusions/Significance:**

These results demonstrate that the innate neutrophil immune response against SAA involves a two-step activation process: an initial signal promoting expression of pro-IL-1β and a second signal involving Syk-dependent activation of the NLRP3 inflammasome and caspase-1, allowing processing of pro-IL-1β and secretion of mature IL-1β.

## Introduction

Serum amyloid A (SAA) is a major acute phase protein produced mainly in the liver, as a result of stimulation by proinflammatory cytokines. SAA also possesses proinflammatory properties that induce the release of cytokines from different cell types, including monocytes [1], [2]. Recent studies showed that SAA induced the expression of pro-IL-1β and activated the NRLP3 inflammasome, resulting in the secretion of mature IL-1β [3]–[5]. IL-1β is a key proinflammatory cytokine with a central role in the damaging inflammatory processes that accompany sterile disease [6]. Caspase-1 is a key protease required for the processing of pro-IL-1β, and its activation is regulated through recruitment to multi-molecular scaffolds called inflammasomes [7]. Inflammasomes are composed of a cytosolic pattern-recognition receptor, pro-caspase-1, and an adaptor molecule [8], [9]. The best characterized inflammasome is NOD-like receptor Pyrin domain containing 3 (NLRP3), which can be activated by a diverse array of disease-associated molecules [10]. It then oligomerizes with the adaptor protein ASC (apoptosis-associated speck-like protein containing a caspase recruitment domain) and caspase-1 to form the NLRP3 inflammasome, which processes pro-IL-1β to mature IL-1β [11]. The NLRP3 inflammasome is activated by various danger-associated molecular patterns, including ATP, monosodium urate (MSU) and aluminum adjuvant [12], [13]. In addition to these stimuli, the NLRP3 inflammasome is also activated by endogenous stimuli including amyloid [14]. SAA exhibits significant immunological activity, including affecting the synthesis of several cytokines, and chemotaxis in neutrophils [15], [16]. It exerts many of its immunological activities by binding to and activating cell surface receptors such as, formyl peptide receptor-like1 (FPRL1) [17], [18]. SAA has also recently been shown to activate the inflammasome cascade, thus highlighting its unique role in immunomodulation [4], [5]. The regulation of IL-1β processing and secretion has been studied extensively in monocytes/macrophages, but the molecular mechanisms leading to IL-1β maturation have not been addressed in neutrophils. We therefore investigated this issue in order to improve our understanding of the role of the inflammasome in the secretion of mature IL-1β by neutrophils.

## Materials and Methods

### Reagents

Recombinant human SAA was purchased from Peprotech (Rocky Hills, NJ). According to the manufacturer, the endotoxin level of the product is 0.1 ng/mg protein. Anti- IL-1β (pro-IL-1β, 3A6), anti-phosho-NF-κB p65 (Ser536) and anti-cleaved caspase-1 (D57A2) antibodies were purchased from Cell Signaling Technology (Beverly, MA). Anti-phosphotyrosine (clone 4G10), anti-Syk (clone4D10) and anti-caspase-1 antibodies were purchased from MERCK MILLIPORE (Billerica,MA USA). Anti-cleaved IL-1β polyclonal antibody was purchased from My BioSource (SanDiego, CA, USA). Caspase-1 inhibitor (Z-YVAD-FMK) was obtained from Abcam (Cambridge, UK). A Syk inhibitor, R406, was purchased from Selleckchem (Houston, Texas USA). Human IL-1β ELISA kit was purchased from R&D systems (Minneapolis, USA).

### Neutrophils Isolation

Venous peripheral blood was collected from healthy volunteers. All participating subjects had given their informed consent. The blood was layered on a Polymorphprep TM (Axis-Shield, Oslo, Norway) cushion and cells were isolated according to the manufacturer’s protocol. Briefly, neutrophils were isolated on the basis of density, washed once in 0.5 N RPMI-1640 to restore osmolality, and then washed once more in RPMI-1640. The PMNs were subsequently diluted in complete medium consisting of RPMI-1640. The study was approved by the Ethics Committees Nagasaki Medical Center and written informed consent was obtained from each individuals.

### Western Blot Analysis

Neutrophils (1×10^6^) were seeded in 24-well plates containing RPMI1640 supplemented with 10% heat-inactivated FBS and stimulated with SAA for 8 hours. Cell-free supernatants were collected by centrifugation at 400 g for 5 minutes. The supernatants or cellular lysates (50 µg) were also subjected to 12% SDS-PAGE, followed by western blot with antibodies against human IL-1β or caspase-1 with an ECL Western blotting kit (Amersham, Little Chalfont, UK).

### Immunoprecipitation Analysis

Neutrophils were pretreated with or without R406 for 1 hr and then stimulated with SAA (5 µg/ml) in serum-free condition. Cells were washed with phosphate-buffered saline, and protein was extracted using lysis buffer (50 mM HEPES, 150 mM NaCl, 1% Triton X-100, 10% glycerol, 1 mM MgCl_2_, 1.5 mM EDTA, pH 8.0, 20 mM β-glycerophosphate, 50 mM NaF, 1 mM Na_3_VO_4_, 10 µg/ml aprotonin, 1 µM pepstatin A, and 1 mM phenylmethylsulfonyl fluoride). After 5 min on ice, the cell lysates were centrifuged at 16,000 g for 10 min at 4°C. The supernatant was saved and the protein concentration was determined using the Bio-Rad protein assay kit (Bio Rad, Hercules, CA). An identical amount of protein (50 µg) for each lysate was subjected to 10% SDS-polyacrylamide gel electrophoresis, and then transferred to a nitrocellulose membrane. Western blot analysis using 4G10 (anti-phosphotyrosine), or phospho-specific anti-ERK1/2, p38, JNK1 antibodies (Cell Signaling Technology) was performed with an ECL Western blotting kit (Amersham, Little Chalfont, UK).

For immunoprecipitation, the cellular lysates were incubated at 4°C with gentle rotation for 3 h in the presence of prewashed protein-G sepharose beads (GE Healthcare, Uppsala, Sweden) linked to an anti-Syk antibody. Protein-G sepharose beads which were then collected and washed three times with cold lysis buffer. Laemmli’s sample buffer (50 µl) was added to the beads, which were boiled for 7 min. The immunoprecipitants were subjected to western blot analysis using 4G10 or anti-Syk antibody.

### Reverse Transcription-Polymerase Chain Reaction (RT-PCR)

Total RNA was extracted from neutrophils using the RNeasy total RNA isolation protocol (Qiagen, Crauley, UK) according to the manufacturer’s protocol. First-strand cDNA was synthesized from 1 µg of total cellular RNA using an RNA PCR kit (Takara Bio Inc., Otsu, Japan) with random primers. Thereafter, cDNA was amplified using specific primers respectively. The specific primers used were as follows:

NLRP3: forward primer 5′- AAAGAGATGAGCCGAAGTGGG-3′ reverse primer 5′- TCAATGCTGTCTTCCTGGCA-3′ β-actin; forward primer 5′-ACGTACATGGCTGGGGTGTTG-3′ reverse primer 5′-GACGAGGCCCAGAGCAAGAGAG-3′.

The product sizes were 79 bp for NLRP3 and 234 bp for β-actin.

The thermocycling conditions (37 cycles) 95°C for 60 s and 63.5°C for 60 s, and 72°C for 60 s.

The amplification of the IL-1βtranscripts was also accomplished on a Light Cycler (Roche Diagnostics, Mannheim, Germany) using specific primers. The housekeeping gene fragment of glyceraldehydes-3-phosphates dehydrogenase (GAPDH) was used for verification of equal loading.

### Statistical Analysis

Differences between groups were examined for statistical significance using Wilcoxon-Mann-Whitney U test. P values less than 0.05 were considered statistically significance.

## Results

### SAA Activates IL-1β Transcription and Processing in Human Neutrophils

**Figure 1:**
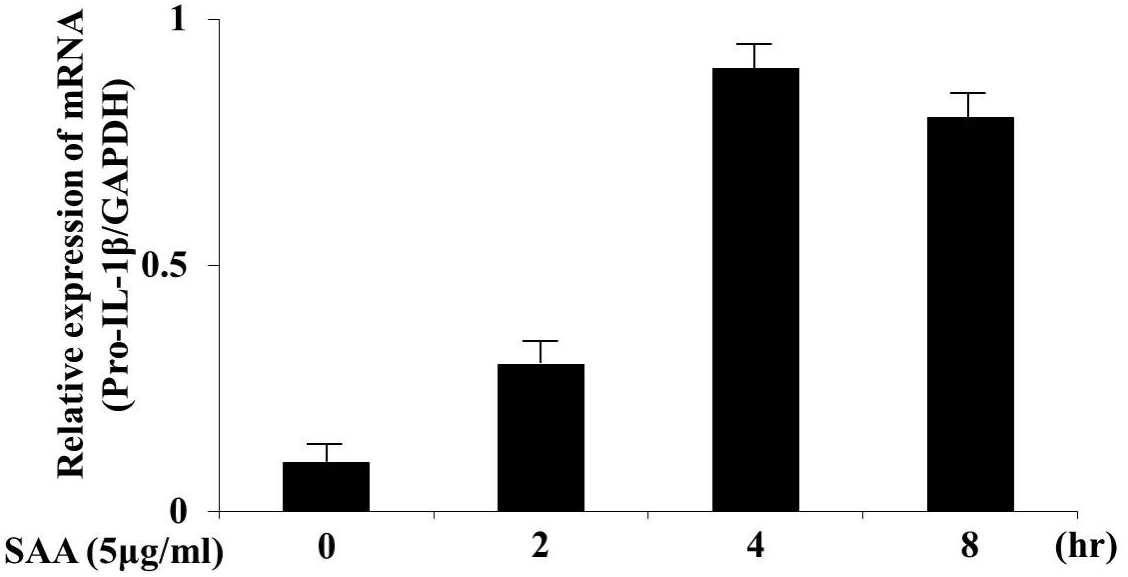
SAA induces the transcription of pro-IL-1β in human neutrophils. Neutrophils were incubated with SAA (5 µg/ml) for the indicated periods. The cells were harvested and analyzed for IL-1β and GAPDH mRNA levels by real-time PCR. Values represent the mean ± SD of three independent experiments.

**Figure 2:**
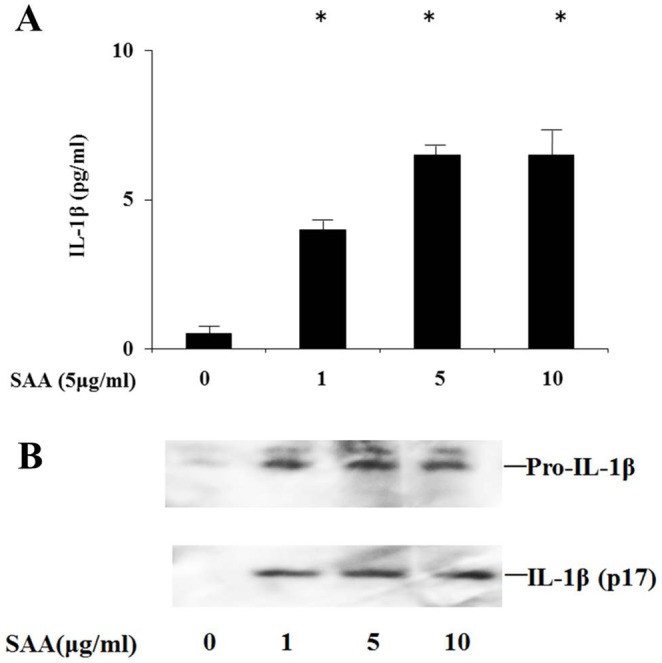
SAA induces mature IL-1β synthesis from neutrophils. **A** Neutrophils were stimulated with the indicated concentrations of SAA for 8-1β production using ELISA. Values represent the mean ± SD of two independent experiments. *p<0.001 compared to SAA-untreated neutrophils. **B** Neutrophils were stimulated with the indicated concentrations of SAA for 8 hr. After stimulation, supernatants were analyzed by western blot analysis for the presence of mature IL-1β. Three experiments were performed using different neutrophils and a representative result is shown.

**Figure 3:**
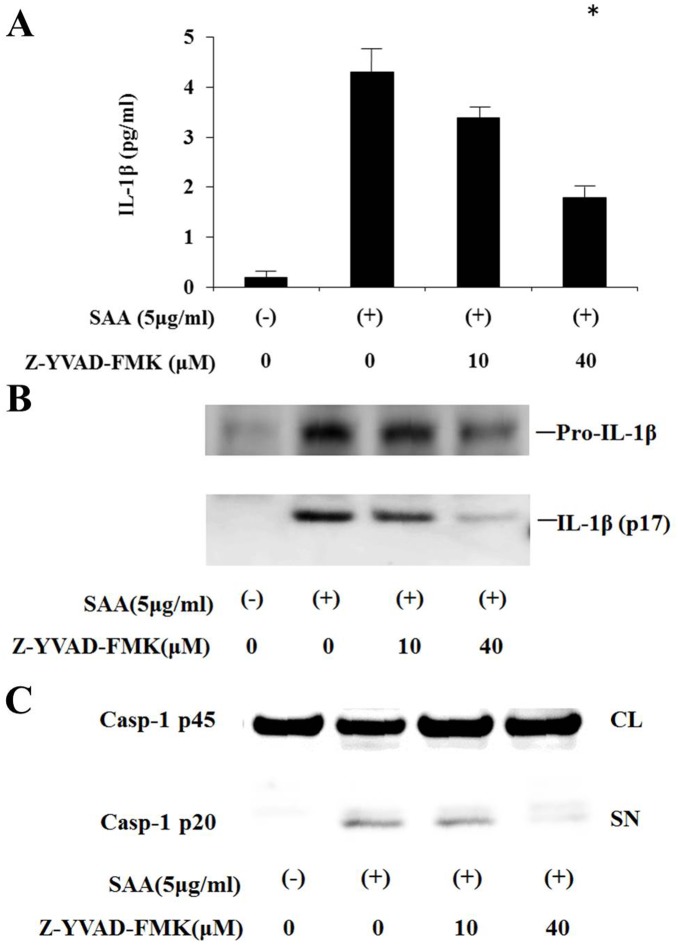
SAA-induced IL-1β processing is dependent on caspase-1. **A** Neutrophils were stimulated with SAA (5 µg/ml) in the presence or absence of Z-YVAD-FMK for 8 h. After stimulation, supernatants were analyzed for IL-1β production using ELISA. Values represent the mean ± SD of two independent experiments. *p<0.001 compared to SAA-stimulated neutrophils. **B** Neutrophils were stimulated with SAA (5 µg/ml) in the presence or absence of Z-YVAD-FMK for 8 h. Supernatants were analyzed by western blot analysis for the presence of mature IL-1β. **C** Neutrophils were stimulated with SAA (5 µg/ml) in the presence or absence of Z-YVAD-FMK for 8 h. Culture supernatants (SN) and cellular lysates (CL) were analyzed by immunoblot using anti-caspase-1 Ab. Caspase-1 (p20, cleaved subunit; p45, precursor). Two experiments were performed using different neutrophils and a representative result is shown.

We examined the proinflammatory cytokine response in human neutrophils stimulated with SAA for different periods. Cells were collected and total cellular RNA was extracted. Pro-IL-1β mRNA expression was studied by real-time PCR. Transcription of the IL-1β gene was strongly activated by SAA at 4 h of SAA stimulation, and then downregulated at 8 h (Figure 1). The transcribed immature IL-1β (pro-IL-1β) needs to be cleaved by caspase-1 to activate secretion of active IL-1β. We investigated if stimulation with SAA resulted in cleavage of pro-IL-1β by determining secreted IL-1β levels in cell culture supernatants after activation by SAA. SAA induced the appearance of the bioactive 17-kDa form of IL-1β in cell culture supernatants, as determined by western blot analysis (Figure 2). These data suggest that SAA triggers both the transcription and secretion of mature IL-1β from neutrophils. The cysteine protease caspase-1 processes pro-IL-1β to biologically active IL-1β [7]. To confirm whether SAA-induced mature IL-1β secretion was dependent on caspase-1-mediated processing of pro-IL-1β, IL-1β secretion was measured in neutrophils activated by SAA in the presence of the specific caspase-1 inhibitor, Z-YVAD. Z-YVAD-FMK prevented SAA-induced IL-1βsecretion and pro-IL-1βprocessing. Additionally Z-YVAD-FMK inhibitor prevented the secretion of activated form of caspase-1, p20, in SAA-stimulated neutrophils (Figure 3).

### R406 Blocks SAA-induced Processing of Pro-IL-1β in Neutrophils

**Figure 4:**
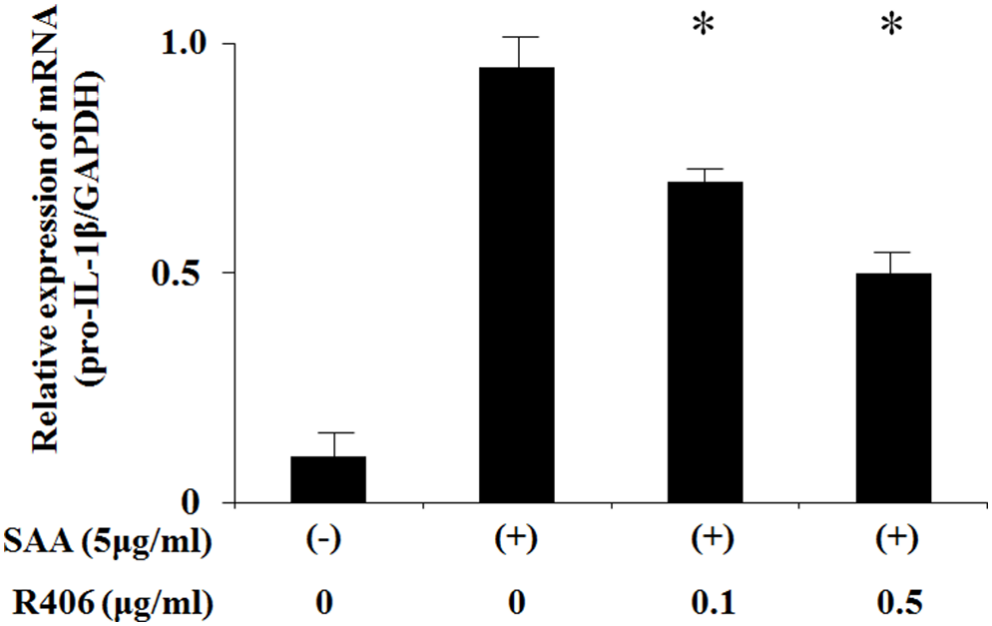
Effects of R406 on the transcription of pro-IL-1β in SAA-stimulated neutrophils. Neutrophils were pretreated or untreated with the indicated concentrations of R406 for 1(5 µg/ml) for 4 hr. The cells were harvested and analyzed for IL-1β and GAPDH mRNA levels by real-time PCR. Values represent the mean ± SD of three independent experiments. *p<0.005 compared to SAA-stimulated neutrophils.

**Figure 5:**
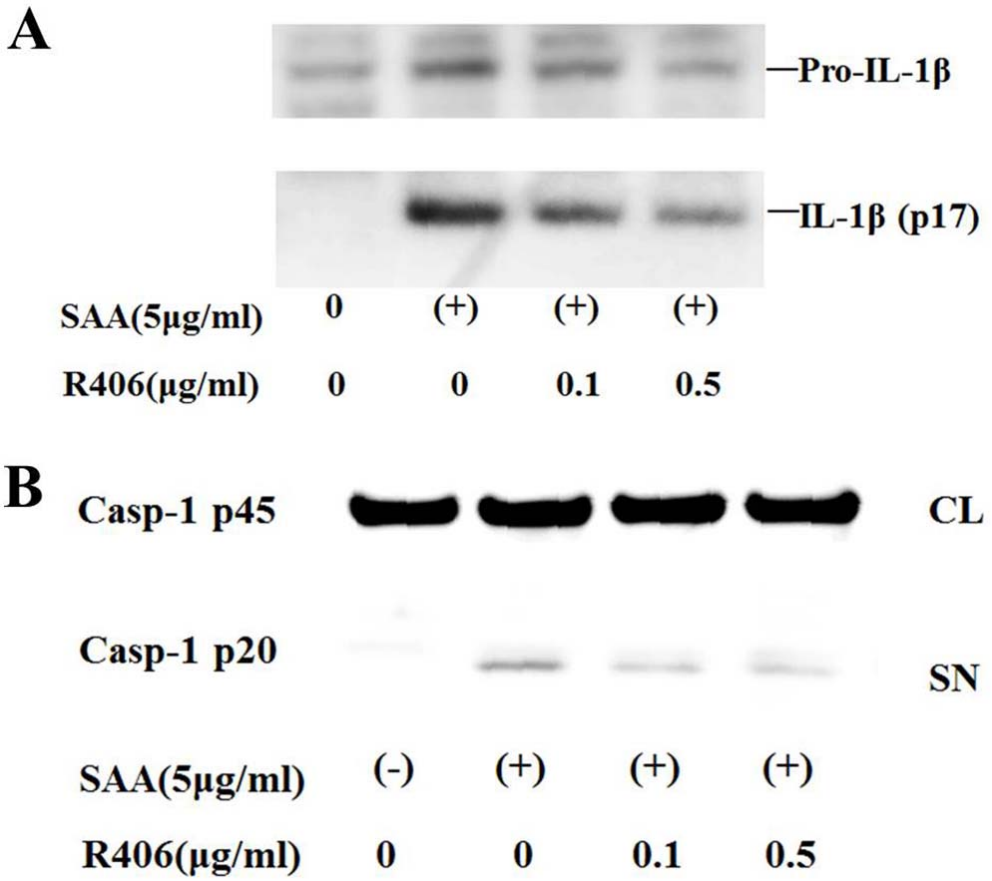
Effects of R406 on SAA-induced IL-1β processing in neutrophils. **A** Neutrophils were pretreated or untreated with the indicated concentrations of R406 for 1(5 µg/ml) for 8 hr. After stimulation, supernatants were analyzed by western blot analysis for the presence of mature IL-1β. Three experiments were performed using different neutrophils and a representative result is shown. **B** Neutrophils were pretreated or untreated with the indicated concentrations of R406 for 1 hr. The cells were stimulated with SAA (5 µg/ml) for 8 hr. After stimulation, culture supernatants (SN) and cellular lysates (CL) were analyzed by immunoblot using anti-caspase-1 Ab. Caspase-1 (p20, cleaved subunit; p45, precursor). Three experiments were performed using different neutrophils and a representative result is shown.

Several lines of evidence suggest that inflammasome activators, such as monosodium urate (MSU) and β-D-glucan, activate the NLRP3 inflammasome via the spleen tyrosine kinase (Syk) signaling pathway [19], [20]. We determined if this signal transducer was required for IL-1β production by analyzing the effects of a specific small molecule Syk inhibitor, R406 [21]. R406 slightly suppressed pro-IL-1β mRNA expression in response to SAA in neutrophils (Figure 4). However, R406 blocked SAA-induced pro-IL-1β processing in neutrophils (Figure 5A). R406 also suppressed the formation of the activated form of caspase-1 p20 in SAA-stimulated neutrophils (Figure 5B).

### SAA Induces NLRP3 Expression in Neutrophils

**Figure 6:**
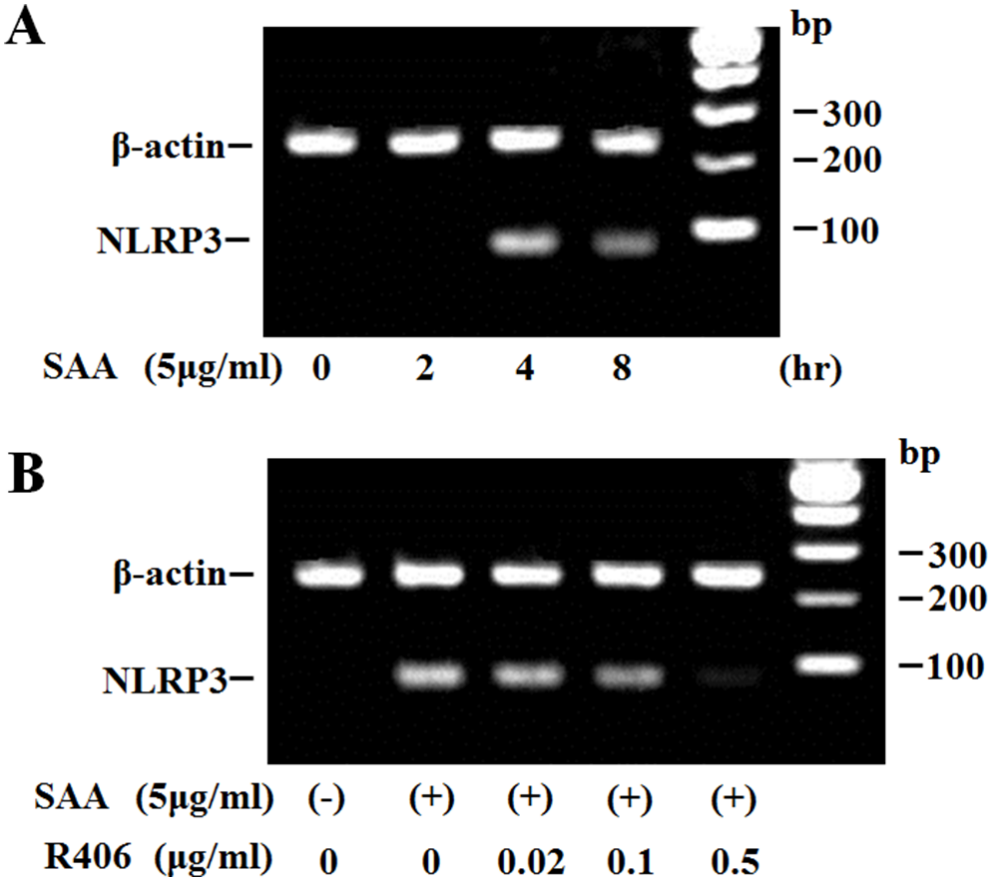
Effects of R406 on the transcription of NLRP3 in SAA-stimulated neutrophils. **A** Neutrophils were pretreated or untreated with the indicated concentrations of R406 for 1(5 µg/ml) for the indicated periods. The cells were harvested and analyzed for NLRP3 mRNA by RT-PCR. **B** Neutrophils were pretreated or untreated with the indicated concentrations of R406 for 1 hr. The cells were stimulated with SAA (5 µg/ml) for 4 hr. The cells were harvested and analyzed for NLRP3 mRNA by RT-PCR. Three experiments were performed using different neutrophils and a representative result is shown.

Induction of NLRP3 expression is required for inflammasome activation. We therefore examined the effects of SAA on NLRP3 expression in neutrophils. *NLRP3* mRNA expression was increased at 4 h in SAA-stimulated neutrophils (Figure 6A). The Syk inhibitor R406 completely prevented SAA-induced *NLRP3* mRNA expression in neutrophils (Figure 6B).

### Effects of R406 on Tyrosine Phosphorylation Response Induced by SAA

**Figure 7:**
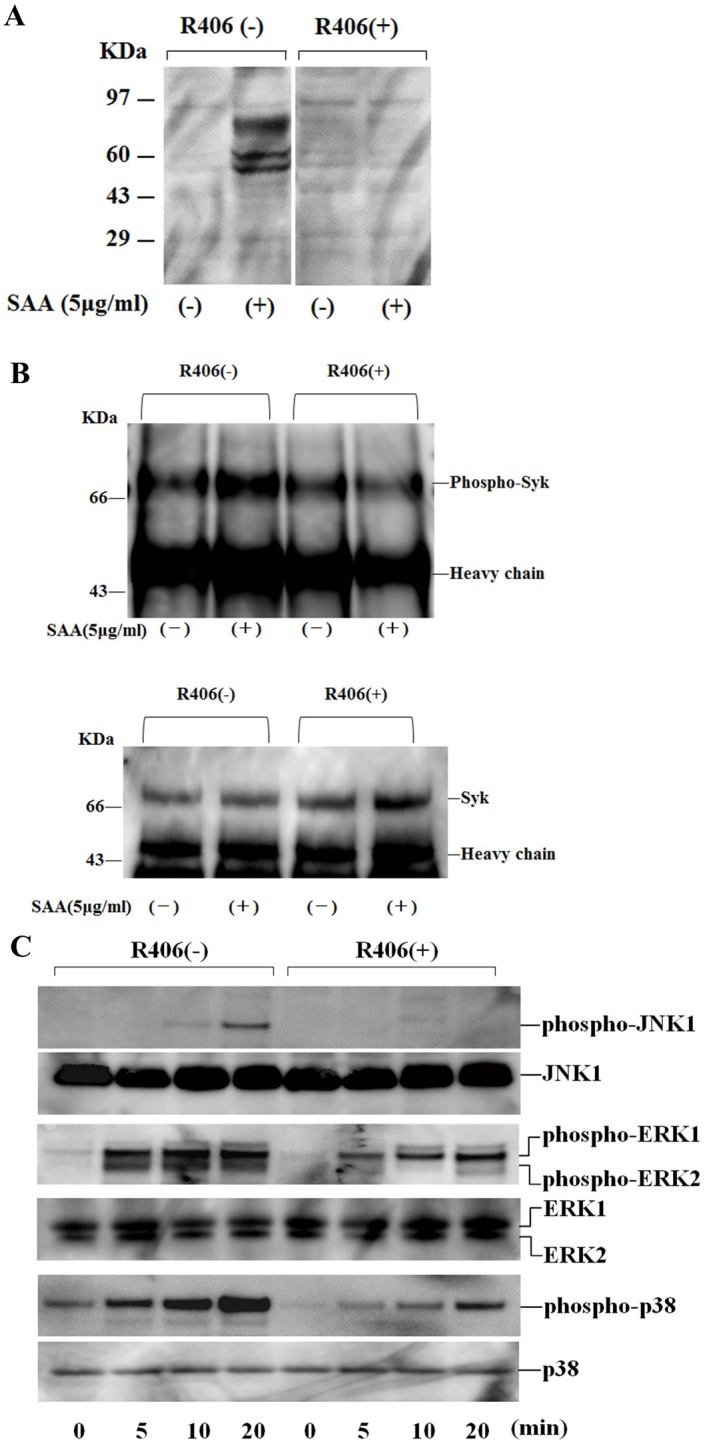
Effects of R406 on the protein phosphorylation in SAA-stimulated neutrophils. **A** Quiescent neutrophils pretreated or untreated with the indicated concentrations of R406 for 1(5 µg/ml) for 20 min. Cellular lysates were subjected to western blotting using phosphotyrosine-specific antibody, 4G10. Three experiments were performed using different neutrophils and a representative result is shown. **B** Quiescent neutrophils pretreated or untreated with the indicated concentrations of R406 for 1 hr. The cells were stimulated with SAA (5 µg/ml) for 20 min. Syk was immunoprecipitated from each lysates and the immunoprecipitates were subjected to western blotting using phosphotyrosine-specific antibody, 4G10 or anti-Syk antibody. Each lane shows Syk precipitated from 10^7^ cells. Three experiments were performed using different neutrophils and a representative result is shown. **C** Quiescent neutrophils pretreated or untreated with the indicated concentrations of R406 for 1 hr. The cells were stimulated with SAA (5 µg/ml). Cellular lysates were subjected to western blotting using phospho-specific or pan antibodies against ERK1/2, p38 and JNK1. Three experiments were performed using different neutrophils and a representative result is shown.

**Figure 8:**
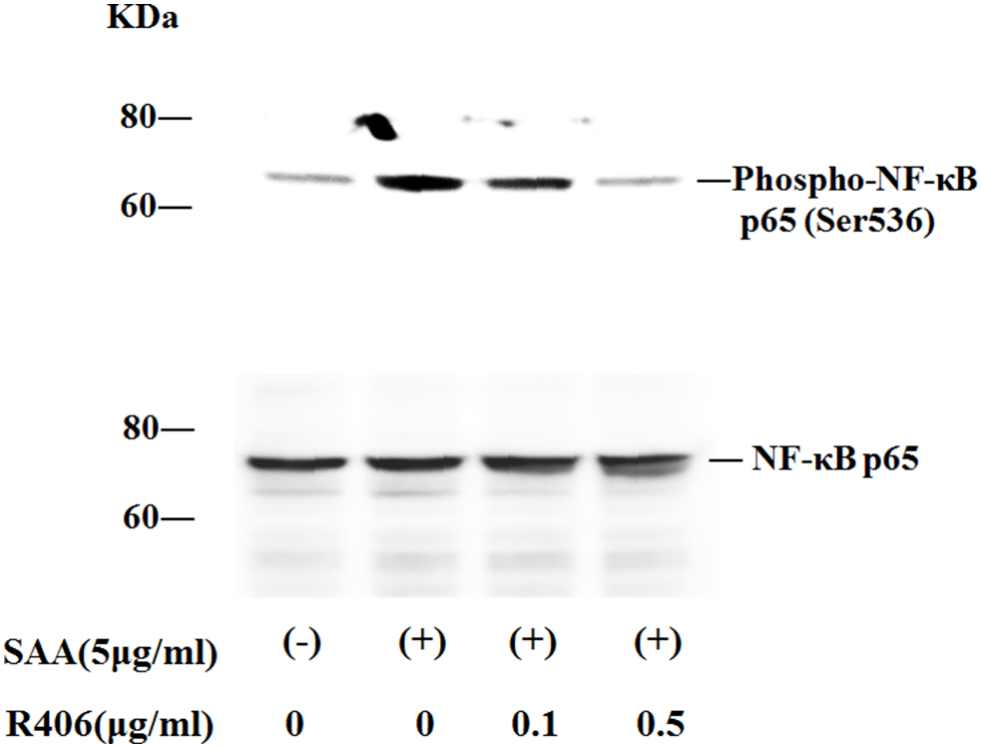
Effects of R406 on NF-κB p65 phosphorylation in SAA-stimulated neutrophils. Neutrophils pretreated with or without R406 were stimulated with SAA (5 µg/ml) for 20 min. Cellular lysates were analyzed by western blotting using anti-phospho-specific p65 (A) or anti-p65 (B) antibodies. Two experiments were performed using different neutrophils and a representative result is shown.

We explored the signaling pathway whereby SAA induces IL-1β production in neutrophils by examining changes in protein phosphorylation profiles in SAA-activated neutrophils. Neutrophils were stimulated with SAA (5 µg/ml) for 20 min. The reactions were stopped by cell lysis, and lysates were analyzed by immunoblotting using anti-phosphotyrosine antibody. The results of a representative experiment are illustrated in Figure 7A. SAA stimulation induced the tyrosine phosphorylation of proteins with apparent molecular masses of 50∼80 kDa. Pre-incubation of neutrophils with R406 inhibited this tyrosine phosphorylation. Syk immunoprecipitation followed by phosphotyrosine analysis revealed that Syk was phosphorylated in response to SAA. SAA-stimulated Syk phosphorylation was blocked by pretreatment with R406 (Figure 7B). We examined whether Syk inhibition affect the downstream signaling pathways, including mitogen-activated protein kinases (MAPKs). Pretreatment with the Syk inhibitor R406 abrogated SAA-induced JNK phosphorylation, while SAA-induced phosphorylation of p38 and ERK1/2 was marginally affected by Syk inhibition with R406. To test whether SAA stimulation induces NF-κB activation in human neutrophils, we examined NF-κB p65 phosphorylation statins in neutrophils. As shown in Figure 8, SAA stimulation induced phosphorylation of p65 as detected by a phosphor-specific Ab that allows monitoring of phosphorylation at serene 536 of p65. R406 pretreatments prevented SAA-induced p65 phosphorylation suggesting that Syk is involved NF-κB activation.

## Discussion

Neutrophils play a crucial role in host defense and the inflammatory response, by virtue of their ability to produce a series of proinflammatory mediators [22], [23]. SAA causes massive infiltration of neutrophils and the secretion of chemokines and cytokines from these cells during inflammation [24]. Direct interactions between SAA and neutrophils thus potentiate activation of the innate immunity that is crucial to the development of inflammation [25]. Previous studies demonstrated SAA-mediated NLRP3 inflammasome activation in monocytes [4]; however, inflammasome activation in neutrophils has not been well-studied. Proteolytic processing of the cysteine proteinase caspase-1 is the hallmark of NLRP3 inflammasome activation [26]. Activated caspase-1 processes pro-IL-1β to the bioactive mature form of IL-1β. Our results revealed that SAA stimulation induced pro-IL-1β mRNA expression in neutrophils and indicated that SAA was capable of processing pro-IL-1β. Consistent with this, the caspase-1 specific inhibitor Z-YVAD-FMK inhibited SAA-induced pro-IL-1β processing. The data presented in this study thus suggest that the endogenous acute phase reactant SAA triggers the IL-1β-mediated inflammatory response in human neutrophils via two separate signals: SAA first binds to neutrophils, activating pro-IL-1β mRNA expression, and then induces NLRP3 mRNA expression resulting in caspase-1-dependent pro-IL-1β processing.

The engagement of FPRLP1 by SAA initiates intracellular signaling that activates nuclear factor-κB and MAPK, leading to the production of proinflammatory cytokines in macrophages [27]. Recent studies defined the roles of dectin-1 and Syk in the activation of the NLRP3 inflammasome, which induces mature IL-1β [28]. However, the role played by endogenous SAA in activating inflammasomes, and its regulation in human neutrophils, have not yet been determined. We defined an essential role for Syk in caspase-1 activation and mature IL-1β secretion in human neutrophils in response to the acute phase reactant, SAA. SAA acts as inducer of NLRP3 mRNA via Syk, and Syk inhibition prevented SAA-induced mature IL-1β secretion in human neutrophils. The SAA-mediated proinflammatory pathway can thus promote the synthesis of mature IL-1β through intracellular signaling molecules in a Syk-dependent manner.

The canonical model of inflammasome activation involves ‘signal 1’ transcriptional upregulation of pro-IL-1β, followed by ‘signal 2’ caspase-1-mediated cleavage of pro-IL-1β into mature IL-1β [29]. The way in which this signaling modulates the inflammasome/IL-1β production remains to be clarified. Our results indicated that Syk inhibition abrogated SAA-mediated NLRP3 expression, suggesting that the Syk pathway is a prerequisite for NLRP3 induction. However, regulation of the inflammasome pathway is probably complex. Like the expression of pro-IL-1β, NLRP3 expression may also affect inflammasome activation. Syk kinase was recently reported to be involved in upstream signaling of the NLRP3 inflammasome triggered by fungi [19]. The adaptor protein, apoptosis associated speck-like protein containing a CARD (ASC), controls inflammasome activity through the activation of caspase-1, and phosphorylation of ASC is critical for speck formation and caspase-1 activation [30]. Recent studies indicate that inhibition of Syk on JNK abolish the formation of ASC speaks. These findings suggest that Syk or JNK-mediated phosphorylation of ASC controls NLRP3 inflammasome activity through the formation of ASC specks[30], [31]. Our data suggest that SAA-induced NLRP3 inflammasome activation in neutrophils is associated with Syk activation, given that the Syk-specific inhibitor R406 resulted in a significant decrease in caspase-1 activation and pro-IL-1β processing. The identification of Syk activation raises the possibility that an unidentified receptor or adaptor protein containing an immunoreceptor tyrosine-based activation motif (ITAM) or ITAM-like domain, such as dectin-1, may also be involved [32]. However, recent work in dendritic cells demonstrated that MSU did not require a surface receptor, and interaction between the crystals and surface lipid rafts was enough to trigger the Syk pathway [33], [34]. Further studies are needed to identify the molecular mechanism whereby SAA interacts with its receptor or intracellular signaling molecule to activate the NLRP3 inflammasome in neutrophils. Gene knock-out studies may help to define the essential components required for the activation of responses by SAA in neutrophils. However, the inability to transfect neutrophils reliably means that the use of chemical Syk inhibitors is unavoidable for examining signaling pathways in human neutrophils.

In conclusion, this study identified a new mechanism of pro-IL-1β processing in neutrophils. These results provide the first evidence demonstrating that the acute phase reactant SAA induces NLRP3 inflammasome activation and IL-1β production via the tyrosine kinase Syk.
